# Pathways among Frailty, Health Literacy, Acculturation, and Social Support of Middle-Aged and Older Korean Immigrants in the USA

**DOI:** 10.3390/ijerph18031245

**Published:** 2021-01-30

**Authors:** Hae Sagong, Ju Young Yoon

**Affiliations:** 1 Nursing Department, College of Nursing, Seoul National University, Seoul 03080, Korea; hsagong@snu.ac.kr; 2 Research Institute of Nursing Science, College of Nursing, Seoul National University, Seoul 03080, Korea; 3 Center for Human-Caring Nurse Leaders for the Future by Brain Korea 21 (BK 21) Four Project, College of Nursing, Seoul National University, Seoul 03080, Korea

**Keywords:** frailty, health literacy, social support, acculturation, older adults, immigrants

## Abstract

Culturally and linguistically different immigrants in the U.S. are considered populations with low health literacy in general, thereby having a high risk of negative health outcomes such as frailty. The purpose of this study is to identify the effects of social support and acculturation on the relationship between health literacy and frailty of Korean immigrants in existing models of health literacy. A total of 244 Korean immigrants aged 50 years and older residing in Southern United States (Alabama and Georgia) were recruited. Path analysis was used to examine the pathways among variables, and the indirect effects of health literacy were analyzed. The results revealed that health literacy and social support directly influenced frailty; social support and acculturation were identified to influence health literacy. Health literacy had a partial mediating effect in the relationship between social support and frailty and a complete mediating effect in the relationship between acculturation and frailty. Therefore, to prevent frailty, it is necessary to consider enhancing immigrants’ health literacy by elevating acculturation and social supports.

## 1. Introduction

Frailty is a state that is vulnerable to stressors with decreased physiological resilience, resulting in increased disability, morbidity, and mortality [1,2]. It is one of the aspects of aging with the largest effect on the deterioration of independence and quality of life in older adults [3]. Fried et al. operationally defined frailty as meeting three or more out of five phenotypic criteria: unintentional weight loss (10 lbs in past year), self-reported exhaustion, weakness (grip strength), slow walking speed, and low physical activity [4]. Previously, research on frailty focused on the physical state but frailty is now used as a predictive tool to identify the decline of practical or social functions in daily life [5,6] along with cognitive, mental, and emotional frailty measurements [7]. Thus, predicting the starting point and progression of frailty in old age is an important index to prevent disease morbidity and poor quality of life. For this reason, the target population for measuring frailty is also expanding from older adults to middle-aged adults. The systematic review of the prevalence of frailty in community-dwelling adults over 65 years of age reported that 10.7% were frail and 41.6% were prefrail [8]; among 49–65 year-old middle-aged community-dwelling African Americans, 2.7% were frail and 37.4% were prefrail, according to the FRAIL scale measurement [9]. Thus, approximately 40% of middle-aged adults can be considered frail or prefrail. The risk factors of frailty were found to be being female, older age, obesity, smoker, and having more than four underlying diseases [2]. Studies found that frailty also appears to increase the risk of morbidity and mortality in middle-aged adults and older adults [2,9]. Therefore, the initial assessment of frailty in middle-aged adults is also important for health management.

Health literacy (HL) is another issue to which older adults are more vulnerable. HL includes not only obtaining and understanding health-related information, but also the ability to appraise and apply health-related information in a sociocultural context to make appropriate health decisions [10]. Low HL affects low medication compliance and self-care ability, health status, and health promotion behaviors, including health screenings [11], resulting in high medical costs, hospitalization periods, and mortality rates [12,13]. However, approximately 36% of American adults had low HL, and older adults over 65 years had a significantly lower level of HL than younger adults [14]. HL does not necessarily mean an individual’s linguistic or knowledge ability but also relates to social aspects such as health-related systems, policies, and endeavors of health-related experts. Therefore, a comprehensive approach is needed to enhance HL [15]. For instance, especially in older adults, it is important to consider contextual factors such as autonomy, supportive social networks, trust in health systems, and easy access to services or information [15]. In addition to older adults, immigrants, minority groups, and low-income groups are also groups with limited HL. Thus, a multifaceted approach is required to enhance HL. Many countries are making efforts to enhance low HL following the characteristics of groups by utilizing various assistive tools, media, social support systems, and approaches that consider the individual’s physical functions [16].

Frailty and HL are particularly important issues for older adults. Recently, several studies have been conducted to examine the relationship between frailty and HL. A study of Taiwanese community-dwelling older adults aged over 65 years determined that HL was correlated with prefrailty and frailty regardless of age, gender, socioeconomic status, and educational attainment [17]. A study conducted in Japan with older adults aged over 60 years revealed that high HL was associated with non-frailty [18]. In contrast, different results were found in the young adults group. The relationships between HL, numeracy literacy, and graph literacy with frailty among male soldiers aged 20 years or older in the U.S. confirmed no evident relationship between HL and frailty [19]. Notable in this study is that despite the average age of the participants being 57 years old, 90% of participants were prefrailty (61.3%) or frailty (28.7%), and 54.9% had inadequate HL [19]. Therefore, frailty and HL should be considered not only for older adults but also for all adult groups. Additionally, the relationship between frailty and HL varied across studies; however, it can be seen that the capacity to understand and apply health-related information affects frailty.

Based on these preceding studies, this study aims to examine the relationship between frailty and HL in middle-aged and older Korean adults who immigrated to the Southern U.S. (Alabama and Georgia). Generally, immigrants are regarded as vulnerable populations with low HL, resulting in serious health disparities and worsening health outcomes [20,21]. Factors affecting the HL of immigrants were language proficiency, educational attainment, gender, age, and poverty level [19]. Of these, language proficiency is a common limitation among most immigrants. Particularly, older immigrants are the most vulnerable group due to limited language proficiency, high demands of physical and mental health, lower income, and issues related to insurance or eligibility status [22]. However, it has been reported that frail older adults obtain health-related information and make medical decisions through the support of close groups, and these social supports were shown to contribute the most important role for them [23]. Since these kinds of social support reduced the negative effects of low HL [24], social factors should be included in the HL interventions and services for older immigrants [25].

Social support played an important role in moderating the negative effects of acculturation stress [26]. Acculturation is generally defined as the psychological adjustment and adaptation to a new culture from another culture [27] and modifications that take place as a result of contact with culturally dissimilar people, groups, and social influences [28]. Most immigrants experience and are affected by adjustment and acculturation throughout their lives. Particularly, low levels of acculturation have been reported to have a significant impact on the deteriorated mental health of older immigrants [26,29,30]. Additionally, higher acculturation and HL levels were associated with an increased likelihood of lifetime and current cancer screening among older immigrants [31]. Studies of immigrants emphasized the importance of social support and acculturation on health; however, most studies are mainly focused on mental health such as depression. Studies on middle-aged immigrants are significantly lacking. Therefore, the purpose of this study was to identify the effects of social support and acculturation on the relationship between HL and frailty in middle-aged and older Korean immigrant adults. The specific questions for this study were as follows: (1) Does HL affect frailty in Korean middle-aged and older immigrants? (2) Do social support and acculturation affect frailty and HL? (3) Does HL have a mediating effect in relationships between other variables? (4) What are the general characteristics that affect HL or frailty?

## 2. Materials and Methods

### 2.1. Study Design and Participants

This was a descriptive cross-sectional survey study to examine the significant paths among HL, social support, acculturation, and frailty. Convenience sampling was used to recruit community-dwelling Korean older adults aged 50 years or older. The sample size was calculated based on the recommendation of a sample to a parameter ratio of 10:1 for testing the mediating effect using structural equation modeling (SEM) [32]. Since the total number of free parameters was 15, including four main variables with four covariates, the minimum sample size was estimated to be 150. The rule of thumb for sample size needed for SEM is more than 200 [32], this study recruited 250 participants considering the dropout. Finally, a total of 244 participants were recruited from the Southern United States (Alabama and Georgia). The inclusion criteria for participants required non-disabled, community-dwelling adults aged 50 years and older who were born in South Korea and had immigrated to the U.S. over a year ago. Participants who could not read, understand, or conduct surveys due to physical or mental illnesses, including deteriorated cognitive function, were excluded. This study was approved by the Institutional Review Board of the Seoul National University (IRB No. 2004/003-022).

### 2.2. Data Collection

Data were collected from June to October of 2020. Surveys given to the participants included questions on general characteristics, measures of frailty, HL, social support, and acculturation. The principal investigator (PI) contacted various Korean centers (e.g., church, company, and community clubs) in Alabama and Georgia, and had a meeting with center directors in person. The PI explained the purpose of the study and, with the consent of the head of the center, began collecting data. Data collection was carried out in three ways: (1) the PI visited the selected centers and directly explained to the participants the nature of the survey, allowed participants to complete their responses followed by the collection of their surveys in a private space away from the PI; (2) participants who requested to complete their surveys at home did so and then submitted their responses to their respective centers; and (3) when the PI was not available, the head of the center explained the purpose of the survey to the participants and then collected their responses. Explanation and written consent preceded all data collected from participants. A gift worth $3 was provided as a sign of gratitude.

### 2.3. Theoretical Framework

The path model of this study was based on a model based on an Intervention Research on Health Literacy among an Ageing Population (IROHLA) comprehensive HL model [15] and an integrated HL model [10]. The comprehensive HL model was developed by Health Literacy Center Europe as part of the IROHLA project. The IROHLA model was developed through a literature review and brainstorming targeting older adults with low HL. The IROHLA model describes interventions such as community support between individuals and experts, empowerment, communication, HL capacity of professionals, and reduction of access barriers to health systems, yield to an increase in HL, and ultimately healthy aging [15].

The integrated HL model was developed through a systematic review of the literature on HL [10]. Accessing, understanding, appraising, and applying health-related information were set as the four competencies of HL, and the determinants of the individual, situational, and social environment were described as antecedents of HL. Personal determinants include general characteristics (e.g., age, gender, income, education attainment, occupation, language, etc.). Situational determinants include social, family, peer support, and the physical environment. Social and environmental determinants include culture, language, and societal systems.

When integrating the above two models, it can be seen that individual, situational, and social and environmental determinants affect HL and ultimately affect frailty. This study designed social support as a situational determinant and acculturation as a social and environmental determinant. Acculturation is a comprehensive concept that refers to a phenomenon that causes changes in cultural patterns when individuals with culturally different backgrounds constantly contact new cultures [33]. In summary, the path model of this study aims to confirm the effects of social support and acculturation on the relationship between HL and frailty, with the personal determinants of middle-aged immigrants as control variables.

### 2.4. Measures

Frailty was assessed using the Korean version of the FRAIL scale [34] proposed by the International Association of Nutrition and Aging [35]. The FRAIL scale includes five components: fatigue, resistance, ambulation, illness, and weight loss. Total frailty scores were calculated by assigning 1 point to positive responses on each of the above five components. Participants with scores of 0, 1–2, and 3–5 were classified as robust, prefrail, and frail, respectively. In path analysis, the total sum score of FRAIL was used as a continuous variable (range: 0–5).

HL was measured using the Health Literacy Survey-12 Questionnaires (HLS-Q12) [36]. HLS-Q12 is a short version of the European Health Literacy Survey Questionnaire (HLS-EU-Q47) [37,38] and has four cognitive domains (access, understand, appraise, and apply health information) with three health domains (health care, disease prevention, and health promotion). HLS-Q12 has a 4-point rating scale ranging from “very difficult” to “very easy” and a higher score indicates higher HL proficiencies (range: 1–4).

Social support was measured using the multidimensional scale of perceived social support (MSPSS) [39]. The MSPSS consists of 12 items with a 7-point Likert scale ranging from “very strongly disagree” to “very strongly agree” and conceptualized with three dimensions: family, friend, and significant others. The MSPSS mean score was used in this study (range: 1–7), and a higher score indicates higher perceived social support.

Acculturation was measured using the East Asian Acculturation Measure (EAAM) [40]. Items were constructed from in-depth semi-structured interviews with East Asian students and faculty regarding social interactions and communication with Americans. The EAAM consists of 29 items with four dimensions: assimilation, separation, integration, and marginalization. Items are scored using a 7-point Likert scale ranging from “strongly disagree” to “agree strongly.” The total score was calculated by summing reversed negative items (separation and marginalization) and positive-scored items (assimilation and integration). In this study, the EAAM mean score was used in analyses (range: 1–7) and a higher score indicates more acculturation.

Covariates were age, gender, monthly income, and length of residency in the U.S. (years) based on previous studies [2,3,10,17,20] to control for the dependent variables in the path analysis model.

### 2.5. Data Analysis

Descriptive analyses were conducted to describe the sample characteristics and the main variables. Correlation analyses were performed to determine the relationships between the four main variables: HL, social support, acculturation, and frailty.

Path analyses were conducted to estimate the pathways among the main variables to examine (1) whether social support or acculturation affects frailty or HL and (2) HL affects frailty. Additionally, indirect effects of HL on the relationship between (1) social support to frailty and (2) acculturation to frailty were confirmed through path analysis. Since path analysis is widely used to examine the mediating effect due to better power and more accurate Type I error rates [41], path analysis is an alternative approach to Baron and Kenny’s traditional method for testing mediating effects. Maximum likelihood estimation with robust standard errors was used in the modeling process considering the non-normal distribution of the main endogenous variables. The Sobel test was used to examine the statistical significance of the indirect effect. The chi-square statistics, root mean square error of approximation (RMSEA), comparative fit index (CFI), Tucker–Lewis index (TLI), and standardized root mean square residual (SRMR) were used to evaluate the model fit [42]. CFI and TLI values above 0.95, RMSEA less than or equal to 0.06, and SRMR less than 0.05 indicate an acceptable fit [42].

Descriptive analyses were completed using SPSS Statistics version 27.0, and path models were estimated using Mplus version 8.3.

## 3. Results

### 3.1. Characteristics of Participants

Table 1 summarizes the characteristics of the participants. The mean age of the 244 participants was 64.05 (SD = 9.46) years. More than half of participants resided in the U.S. for more than 15 years (55.7%). The total mean score of acculturation was 3.83 (SD = 0.66). Among the acculturation dimensions, separation was the highest (mean score 5.14, SD = 0.94) and marginalization was the lowest (mean score 3.15, SD = 0.95). The mean scores of social support and HL were 5.32 (SD = 1.02) and 2.55 (SD = 0.59), respectively. The percentages of participants’ frail status, robust, prefrail, and frail were 58.6%, 35.7%, and 5.7%, respectively. The Cronbach’s *α* of the three main variables in this study, acculturation, social support, and HL were 0.84, 0.94, and 0.95, respectively.

### 3.2. Path Analysis

Prior to conducting path analysis, correlations among the main variables were confirmed (Table 2). The results identified significant correlations in which acculturation and social support with HL were positively correlated (*r* = 0.51 and *r* = 0.42, respectively) and frailty was negatively correlated with social support, acculturation, and HL (*r* = −0.25, *r* = −0.23, and *r* = −0.28, respectively).

**Figure 1:**
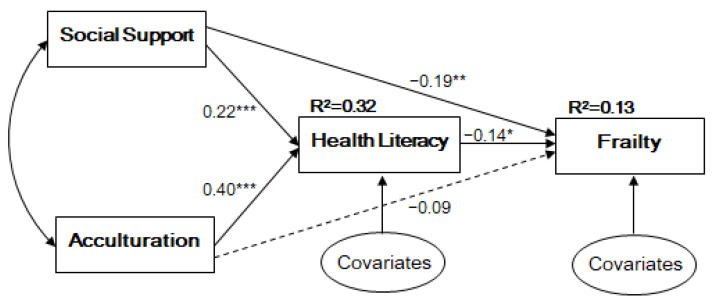
Path Model. Note. * *p* < 0.05, ** *p* < 0.01, *** *p* < 0.001 All coefficients are standardized; Covariates are age, gender, income, immigration period; Model fit indices: χ^2^ (2) = 2.144 (*p* = 0.342), RMSEA = 0.017, CFI = 0.999, TLI = 0.991, SRMR = 0.020.

Table 3 and Figure 1 present the significance of the model. Acculturation (*β* = 0.40, *p* < 0.001) and social support (*β* = 0.21, *p* < 0.001) exhibited a significant and positive relationship with HL. For frailty, HL (*β* = −0.14, *p* = 0.032) and social support (*β* = −0.19, *p* = 0.009) had a significant, negative, and direct relationship. Among the covariates, the immigration period was significantly related to HL (*β* = 0.16, *p* = 0.003). This path model demonstrated a good model fit (CFI = 0.999, TLI = 0.994, RMSEA = 0.014, SRMR = 0.020), and explained 32% and 13% of the variance of HL and frailty, respectively.

### 3.3. Mediating Effect of Health Literacy

The mediating effect of HL is presented in Table 4. HL had a significant partial mediating effect (*β* = −0.03, *p* = 0.045) in the relationship between social support and frailty. In the relationship between acculturation and frailty, HL had a significant complete mediating effect (*β* = −0.06, *p* = 0.048).

## 4. Discussion

This study demonstrated that HL and social support directly influenced frailty and social support and acculturation influenced HL. Furthermore, HL had a partial mediating effect in the relationship between social support and frailty and a complete mediating effect in the relationship between acculturation and frailty. Although the indirect effect of HL was relatively small, it is notable that frailty can be mediated by HL.

Several studies have found a relationship between frailty and HL [17,18,19], but these studies were conducted in their own country with the same nationality and only confirmed the relationship between HL and frailty. This study has an implication that it was conducted with minority ethic immigrants in the U.S. who have a totally different cultural and linguistic context. By controlling the general characteristics of immigrants that affect HL or frailty (e.g., gender, age, income, and length of residency in the U.S.), HL had a significant effect on frailty, indicating that lower HL is associated with increased frailty. Since immigrants are regarded as one of the most venerable minority populations with low HL [20,21], they are considered to be at high risk of frailty. However, in a study conducted with middle-aged and older immigrants in Europe, the level of frailty was affected by the country of origin/birth and current residence in the country [43]. Therefore, studies examining the relationship between HL and the frailty of immigrants with various ethnicities and regions are needed.

Social support exhibited a significant role in this study by affecting HL and frailty. Older adult immigrants tended to depend on social support from the same nationality groups of the community or their family. Particularly, family members play a major role in their health care decision-making [20]. As evident from this study, due to their characteristics of having trouble adapting to a new host country, social support was positively associated with HL, which can be interpreted as social support contributing to lessening the negative effects of low HL. This result is consistent with previous studies demonstrating that low social support was significantly associated with poor HL [24,44] and low overall health status [45]. Particularly, social support was significantly and positively associated with the overall health status in the HL group [45]. Studies verifying the mediating effect of HL on the relationship between social support and frailty are lacking; therefore, further research is required to verify the results of this study. However, a key point is that social support should be considered significantly to enhance HL and the health status of older immigrants.

Longer residency in the U.S. affected higher HL, meaning that immigrants who have lived in the U.S. for a long time are better able to adapt to their host culture and language, thereby having an increased ability to acquire health-related information. This result was consistent with the pathway of acculturation to HL, in which the meaning of acculturation is one’s adaptation to a new culture. Acculturation exhibited a significant effect on frailty through HL but showed no significant direct effect on frailty in this study. Therefore, to reduce the risk of frailty, there is a need for strategies to improve the immigrants’ level of acculturation to induce a positive effect on HL. Participants in this study scored higher in the separation dimension (Table 1), while Asian young adults with a mean age of 28.7 years with 7.4 years of living in the U.S. scored higher in the integration dimension [40]. Even though more than half of participants in this study lived in the U.S. for more than 15 years (55.7%), they still maintained their heritage identity and traditions and did not have relations with the new host country [40]. The characteristics of acculturation should also be considered as interventions to enhance HL.

Since the U.S. has a larger immigrant population than any other country, with 44.8 million consisting of 13.7% of the U.S. population [46], health systems should focus on enhancing HL to induce positive health outcomes. In addition, health policy and experts should account for the characteristics of older immigrants that they mainly rely on their social network when making health-related decisions and culturally separated to the host country.

### Limitations

Since this study was conducted on Korean immigrants residing in several states in the Southern U.S. with convenience sampling, there is a limitation of generalizability of the results. Considering that significant pathways may appear different across different age groups, it is necessary to examine with age groups of sufficient samples and verify as an individual model. In addition, this study measured frailty with a self-reported scale. The prevalence of frailty in this study was similar to that of previous studies; however, a physical measurement for frailty is also required to validate this study. Finally, this study was a cross-sectional study showing only the relationship between variables. A longitudinal study is recommended to investigate causality.

## 5. Conclusions

This study had an implication that revealed relationships among frailty, HL, social support, and acculturation of middle-aged and older Korean immigrants in the U.S. based on two integrated models with path analysis. Social support and HL exhibited significant effects on frailty while acculturation did not, and HL had an indirect effect on the relationship between frailty and social support, frailty, and acculturation, respectively. Therefore, to prevent frailty, it is necessary to consider enhancing immigrants’ health literacy by elevating acculturation and social supports.

## Figures and Tables

**Table 1:** Characteristics of the participants (*N* = 244).

Variables	Category	*N* (%) or M ± SD
Age	Total	64.05 ± 9.46
	50–59	96 (39.3)
	60–69	71 (29.1)
	70≤	77 (31.6)
Gender	Male	119 (48.8)
	Female	125 (51.2)
Length of residency (years)	<5	4 (1.6)
	5≤~<10	44 (18.0)
	10≤~<15	60 (24.6)
	15≤	136 (55.7)
Educational attainment	Middle	16 (6.6)
	High	72 (29.5)
	Bachelor	114 (46.7)
	Graduate	42 (17.2)
Monthly income (dollars)	None	17 (7.0)
	<1000	34 (13.9)
	1000≤~<3000	65 (26.6)
	3000≤~<5000	64 (26.2)
	>5000	63 (25.8)
Acculturation (range: 1–7)	Total	3.83 ± 0.66
	Assimilation	2.25 ± 1.08
	Separation	5.14 ± 0.94
	Integration	3.29 ± 1.16
	Marginalization	3.15 ± 0.95
Social support (range: 1–7)		5.32 ± 1.02
Health literacy (range: 1–4)		2.55 ± 0.59
Frailty	Robust	143 (58.6)
	Prefrail	87 (35.7)
	Frail	14 (5.7)

**Table 2:** Correlation coefficient of variables (*N* = 244).

Variable	Acculturation	Social Support	Health Literacy	Frailty
Acculturation	1.00	0.32 ***	0.42 ***	−0.25 ***
Social support		1.00	0.51 ***	−0.23 ***
Health literacy			1.00	−0.28 ***
Frailty				1.00

Note. *** *p* < 0.001.

**Table 3:** Path estimates and significance of the model.

Model Pathway			*β*	S.E.	*p*
Health literacy	←	Acculturation	0.40	0.06	<0.001
		Social support	0.22	0.05	<0.001
		Gender	0.03	0.05	0.518
		Length of residency	0.16	0.05	0.003
Frailty	←	Health literacy	−0.14	0.07	0.032
		Acculturation	−0.09	0.08	0.253
		Social support	−0.19	0.07	0.009
		Age	0.12	0.08	0.149
		Gender	−0.05	0.07	0.472
		Income	0.08	0.07	0.280
		Length of residency	0.08	0.07	0.278
Model fit indices		χ2 (2) = 2.144 (*p* = 0.342), RMSEA = 0.017, CFI = 0.999, TLI = 0.991, SRMR = 0.020			

Note. Values are standardized coefficients; S.E. = standard error; RMSEA = Root Mean Square Error of Approximation; CFI = Comparative Fit Index; TLI = Tucker-Lewis Index; SRMR = Standardized Root Mean Square Residual.

**Table 4:** The mediating effect of health literacy.

Model Pathway	Effect	*β*	S.E.	*p*
Frailty ← Social support	Total	−0.22	0.07	0.003
	Indirect	−0.03	0.02	0.045
	Direct	−0.19	0.07	0.009
Frailty ← Acculturation	Total	−0.15	0.07	0.038
	Indirect	−0.06	0.03	0.048
	Direct	−0.09	0.08	0.253

Note. All coefficients are standardized; S.E. = standard error.
